# Endoplasmic Reticulum-Mitochondria Crosstalk and Beta-Cell Destruction in Type 1 Diabetes

**DOI:** 10.3389/fimmu.2021.669492

**Published:** 2021-04-16

**Authors:** Saurabh Vig, Joost M. Lambooij, Arnaud Zaldumbide, Bruno Guigas

**Affiliations:** ^1^Department of Cell and Chemical Biology, Leiden University Medical Center, Leiden, Netherlands; ^2^Department of Parasitology, Leiden University Medical Center, Leiden, Netherlands

**Keywords:** endoplasmic reticulum, ER stress, mitochondria, Type 1 diabetes (T1D), beta-cell, inflammation, cytokines

## Abstract

Beta-cell destruction in type 1 diabetes (T1D) results from the combined effect of inflammation and recurrent autoimmunity. In response to inflammatory signals, beta-cells engage adaptive mechanisms where the endoplasmic reticulum (ER) and mitochondria act in concert to restore cellular homeostasis. In the recent years it has become clear that this adaptive phase may trigger the development of autoimmunity by the generation of autoantigens recognized by autoreactive CD8 T cells. The participation of the ER stress and the unfolded protein response to the increased visibility of beta-cells to the immune system has been largely described. However, the role of the other cellular organelles, and in particular the mitochondria that are central mediator for beta-cell survival and function, remains poorly investigated. In this review we will dissect the crosstalk between the ER and mitochondria in the context of T1D, highlighting the key role played by this interaction in beta-cell dysfunctions and immune activation, especially through regulation of calcium homeostasis, oxidative stress and generation of mitochondrial-derived factors.

## Introduction

Type 1 diabetes (T1D) is an autoimmune disease that targets pancreatic beta-cells, leading to their progressive loss (1). For years, impaired thymic education or low affinity T cells were thought to be responsible of the immune attack directed against native self-proteins. However, accumulating evidence suggest that local inflammation or other forms of stress, like viral infection, toxic chemical exposure or dysglycemia, combined with genetic predisposition may lead to the generation and accumulation of aberrant or modified proteins to which central tolerance is lacking, thereby triggering autoimmunity (2–4).

The endoplasmic reticulum (ER), is the hub for protein synthesis, folding, modification and transport as well as for phospholipid and cholesterol biosynthesis (5). Alterations in ER homeostasis due to inflammatory stress, accumulation of misfolded proteins, and/or alterations in the cellular Ca^2+^ or redox balance triggers an unfolded protein response (UPR) through activation of ER transmembrane proteins [*e.g.* double-stranded RNA-activated protein kinase (PKR)-like ER kinase (PERK), inositol-requiring enzyme 1α (IRE1α) and activating transcription factor 6 (ATF6)]. These central mediators of the UPR sense the accumulation of misfolded proteins in the ER lumen and activate mechanisms to inhibit protein synthesis, restore expression of chaperones, like the 78-kDa glucose regulated protein [also known as binding immunoglobulin protein] (GRP78/BiP), and initiate ER associated degradation pathway to eliminate newly synthesized proteins through proteasome-mediated degradation (6, 7). Persistent stimulation of the UPR in response to ER stress induces apoptosis *via* activation of C/EBP homologous protein (CHOP), c-jun N-terminal kinase (JNK), death protein 5 (DP5) and other pro-apoptotic signals (8, 9). Several studies, have demonstrated that this adaptive phase disturbs (post)-transcriptional, (post)-translational and degradation processes, increasing the complexity of the beta-cell proteome and peptidome, promoting the generation of neoantigens (10, 11).

Like the ER, mitochondria are complex and dynamic cellular organelles that play a key role in beta-cell functions, notably by coupling glucose metabolism to insulin secretion, but also in regulating apoptotic cell death *via* the production of reactive oxygen species (ROS) and release of cytochrome C (12, 13). In most eukaryotic cells, including beta-cells, mitochondria form dynamic networks that are continually reshaped by fission and fusion processes, under the control of specific mitochondrial membrane anchor proteins. Induction of the mitochondria UPR (UPRmt) plays an essential role in the maintenance of the mitochondrial integrity, dynamics and function in response to various stressors (14, 15). Currently, little is known regarding the impact of pro-inflammatory stimuli on mitochondrial dynamics/bioenergetics and UPRmt in human beta-cells. Yet, the interaction between the ER and mitochondria during the adaptive mechanism to environmental stress indicates that both organelles orchestrate the communication between the beta-cells and the immune system. Therefore, further exploring the regulatory mechanisms involved in mitochondria-ER interaction and in particular those controlling Ca^2+^ homeostasis and mitochondrial homeostasis, is required for a better understanding of the pathophysiology of beta-cell failure and its immune-related consequences in T1D.

## ER-Mitochondria Crosstalk in Beta-Cell (dys)Functions

The ER and mitochondria are organelles that physically interact in a highly dynamic and regulated manner, forming specific microdomains, termed mitochondria and ER contact sites (MERCs) or mitochondria-associated membranes (MAMs) when studied at the molecular level (16). It is well established that MAMs play a central role in cellular Ca^2+^ homeostasis (17–19) and, more recently, they have also been shown to regulate mitochondrial dynamics and bioenergetics (20), ROS production (21), mitochondrial-mediated apoptosis (22), and inflammation (22, 23). MAMs are composed of membrane fractions from both the ER and the outer mitochondrial membrane (OMM) containing a large range of cell-specific molecular components involved in the tethering complex (16). Alterations in the MAMs composition and abnormal ER-mitochondria interaction have been reported to be associated with different pathological conditions, especially in type 2 diabetes (T2D) where organelle miscommunication has been suggested to underlie beta-cell inflammation, cell death and impaired metabolic function (24).

### ER-Mitochondria Tethering, Ca^2+^ Homeostasis and Beta-Cell Dysfunction

**Figure 1:**
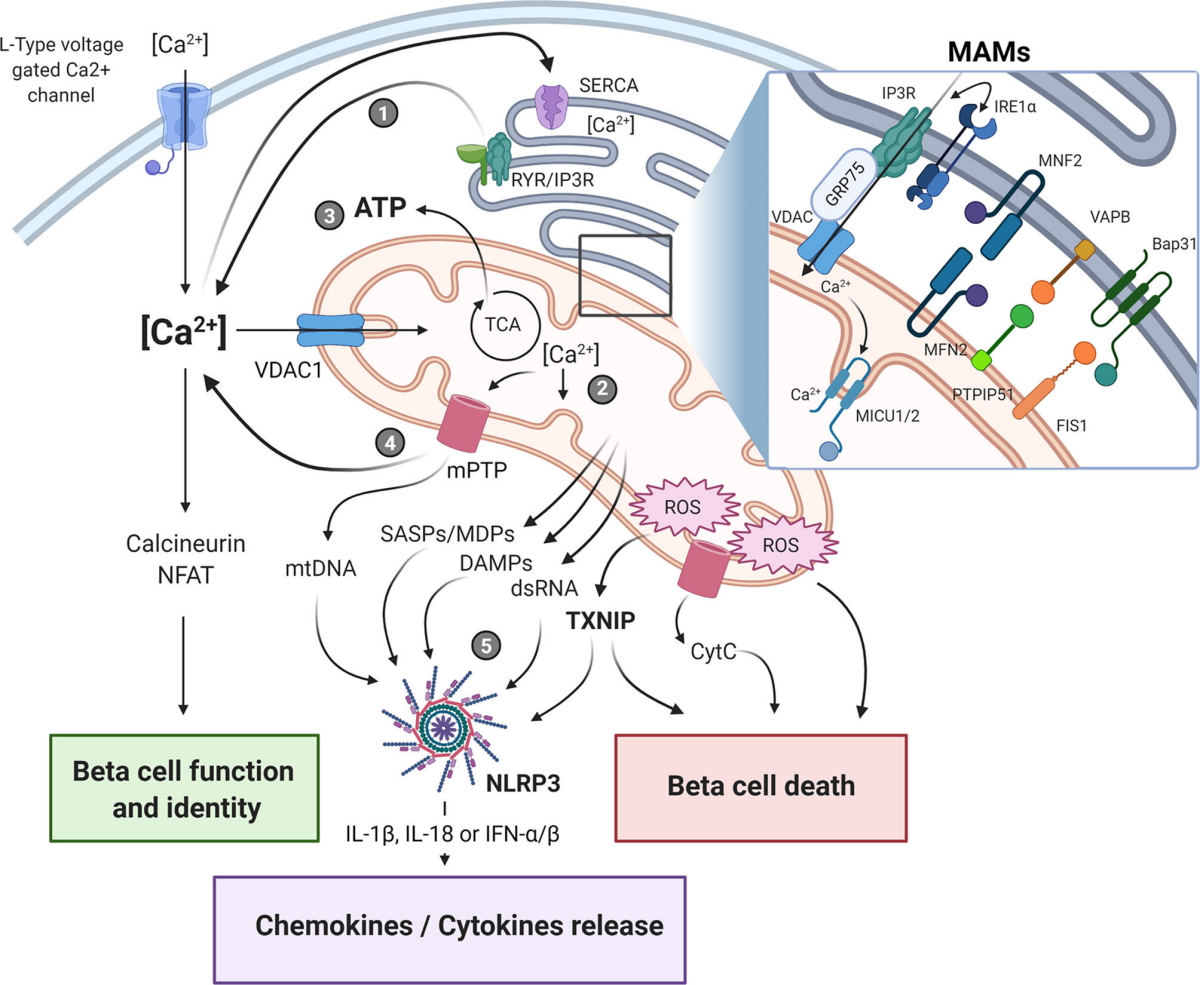
Endoplasmic reticulum-mitochondria crosstalk in beta-cell dysfunction and immune activation in type 1 diabetes. The crosstalk between the endoplasmic reticulum (ER) and the mitochondrial network is playing a central role in cellular Ca^2+^ homeostasis and beta-cell function. The physical ER-mitochondria interaction involves a set of proteins located in the mitochondrial-associated membranes (MAMs, insert) which allows the exchange of organelle respective content. Modulation of MAM assembly has been shown to regulate mitochondrial dynamics and bioenergetics, ROS production, and release of various mitochondrial-derived factors. Alterations of these highly regulated processes in response to ER/mitochondria stress, can contribute to beta-cell dysfunction and immunogenicity by triggering apoptotic beta-cell death and NLRP3 activation, ultimately promoting pro-inflammatory cytokine/chemokine production, immune cells recruitment within the islet microenvironment, and beta-cell destruction. Upon ER stress induction, the depletion of ER Ca^2+^ store (1) induces mitochondrial Ca^2+^ accumulation (2). This increase in mitochondrial matrix Ca^2+^ concentration triggers oxidative phosphorylation and ATP production (3) in order to restore cellular and ER homeostasis. If this adaptive response fails to restore homeostasis, prolonged Ca^2+^ accumulation in the mitochondria would promote the opening of the mPTP, leading to increased mitochondrial membrane permeability and leakage of mitochondrial-derived factors, such as DAMPS, mtDNA, dsRNA (4) that ultimately activate downstream sensors/inflammasome (5). SASPs, senescence-associated secretory phenotype; DAMPs, damage-associated molecular patterns; MDPs, mitochondrial-derived peptides; ROS, reactive oxygen species; mtDNA, mitochondrial DNA; mPTP, mitochondrial permeability transition pore; CytC, cytochrome C. The figure was created with BioRender.com.

The regulation of Ca^2+^ homeostasis is essential for proper beta-cell functions, because of its role in driving insulin granule biogenesis, trafficking and exocytosis but also by triggering multiple intracellular signaling pathways essential for the maintenance of beta-cell identity and survival (25). Cytosolic Ca^2+^ concentration is tightly controlled and results from a balance between its cellular influx and efflux, and its intracellular uptake and release by various organelles, such as ER, Golgi and the mitochondria, through specific exchangers, pumps, and channels (**Figure 1**). It is still unclear whether the mitochondria can play a significant role in directly buffering cytosolic Ca^2+^ in a quantitative manner under physiological conditions (26). However, acute and/or long-lasting modulation of inter-organelle communication, particularly under pathological conditions, may impact Ca^2+^ homeostasis in beta-cells. As such, channeling of the cation in between subcellular compartments, notably from the ER to the mitochondria, represents another way by which large quantities of Ca^2+^ can be conveyed and exert key regulatory roles on the organelle functions. Under homeostatic conditions, a transient increase in beta-cell mitochondrial matrix Ca^2+^ levels promotes ATP production by oxidative phosphorylation (OXPHOS). This occurs principally through direct activation of several tricarboxylic acid (TCA) cycle dehydrogenases and contributes to K_ATP_ channel-mediated opening of L-type voltage-gated Ca^2+^ channels (L-VGCCs), increased cytosolic Ca^2+^ and sustained glucose-stimulated insulin secretion (GSIS) (27). However, any perturbations of this highly regulated spatio-temporal process would result in an altered mitochondrial homeostasis that may ultimately lead to bioenergetic dysfunction, enhanced oxidative stress and cell death. The ER-mitochondrial connectivity was shown to involve a set of interacting proteins located in the MAMs (**Figure 1****, **insert****), allowing ER and mitochondria to share their content, especially Ca^2+^, through the 75-kDa glucose-regulated protein (GRP75)-mediated coupling of the ER inositol trisphosphate receptor (IP3R) with the mitochondrial voltage-dependent anion-selective channel 1 (VDAC1) (28). The association formed by the ER vesicle-associated membrane protein-associated protein B (VABP) with the OMM protein tyrosine phosphatase-interacting protein-51 (PTPIP51) could also contribute in the organelle interaction (29). Moreover, proteins involved in mitochondrial dynamics were also shown to be involved in this tethering, such as mitofusin 2 (MFN2) and mitochondrial fission 1 protein (FIS1). Indeed, FIS1 can directly interact with the ER B-cell receptor-associated protein 31 (Bap31), promoting Ca^2+^ transfer from ER to the mitochondria and subsequent induction of apoptosis (30).

Interestingly, it has recently been reported that besides its canonical function in UPR, IRE1α also acts as a scaffold within the ER-mitochondria junction by directly interacting with IP3R, the main ER Ca^2+^ channel, ultimately affecting ER-mitochondria Ca^2+^ signaling (31). Indeed, the interaction between the respective C-terminal cytosolic domains of monomeric IRE1α and IP3R proteins within the MAMs allows for a VDAC1-mediated transfer of Ca^2+^ from the ER lumen to the mitochondrial matrix, leading to increased Ca^2+^ concentrations that trigger activation of TCA cycle and ATP production (31). Interestingly, it has been shown in pancreatic tissue sections that the number of IP3R-VDAC1 complexes assessed by *in situ* proximity ligation assay was significantly lower in beta-cells from individuals with T2D as compared to non-diabetic controls, suggesting that alterations in the ER-mitochondria interaction may contribute to beta-cell dysfunction under diabetic conditions (32). Furthermore, induction of ER stress by treatment with the long-chain fatty acid palmitate significantly reduced ER-mitochondria interactions and altered GSIS in the murine Min6-B1 beta-cell line (32). More recently, the same team has also reported that both acute or prolonged exposure to glucose may promote ER-mitochondria interactions while having opposite impact on mitochondrial Ca^2+^ homeostasis and beta-cell insulin secretion (33). Indeed, it was shown that an acute glucose challenge enhanced MAMs assembly, as evidenced by increased VDAC1-IP3R2 proximity, which was associated with higher ATP-stimulated ER-mitochondrial Ca^2+^ transfer, intramitochondrial Ca^2+^ accumulation and GSIS in rat INS-1E beta-cell line. This ER-mitochondria interaction was abolished when MAM tethering protein GRP75 was knocked down by siRNA, demonstrating that organelle coupling is crucial for beta-cell Ca^2+^ homeostasis and insulin exocytosis under homeostatic conditions (33). Counterintuitively, extending the duration of glucose exposure to mimic diabetogenic conditions also resulted in increased MAM formation in both INS-1E cells and human pancreatic islets. However, despite higher organelle interactions, chronic glucose exposure triggered ER stress and disruption of ER-mitochondrial Ca^2+^ exchange, characterized by a progressive depletion of the ER Ca^2+^ store and increased Ca^2+^ levels in fragmented mitochondria, ultimately leading to altered mitochondrial respiration and impaired GSIS (33). Remarkably, this feature also resembles the effect of pro-inflammatory cytokines in the context of T1D, with Ca^2+^ depletion in the ER being associated with enhanced protein misfolding, UPR activation, and beta-cell death (34). Of note, treatment with the ER stress inducer tunicamycin also increased VDAC1-IP3R2 interactions in INS-1E cells, suggesting that increased organelle tethering could constitute an adaptive mechanism for restoring ER-mitochondria Ca^2+^ homeostasis (33). Whether beta-cell ER stress and IRE1α-mediated UPR associated with islet inflammation in T1D would be the cause or the consequence of impaired MAM assembly that leads to altered mitochondrial Ca^2+^ homeostasis and mitochondria-mediated immune activation, remains however to be investigated.

### ER-Mitochondria Crosstalk, Oxidative Stress and Beta-Cell Death

MAMs have also been identified as a critical hub in the regulation of beta-cell death, because ER stress and alterations in mitochondrial Ca^2+^ homeostasis are linked to increased local ROS production and induction of intrinsic apoptotic pathways (35, 36). IRE1α was shown to be involved in the regulation of the OMM B cell lymphoma-2 (Bcl-2) protein family. Once activated, IRE1α can bind the TNF receptor associated factor 2 (TRAF2) adaptor and activate the JNK signaling pathway, triggering apoptosis *via* upregulation of CHOP and downregulation of the anti-apoptotic protein Bcl-2 (10). Additionally, CHOP promotes Bcl-2 phosphorylation which impairs its inhibitory interaction with the pro-apoptotic proteins, Bcl-2 associated X protein (Bax) and Bcl-2 homologous antagonist killer protein (Bak). These proteins induce IP3R-mediated cytosolic release of Ca^2+^ from the ER which is next channeled into the mitochondria through VDAC1 (37, 38). This causes an increase in mitochondrial matrix Ca^2+^ concentration that triggers the opening of the mitochondrial permeability transition pore (mPTP), a non-specific channel located in the inner mitochondrial membrane (IMM), ultimately leading to cytochrome C-mediated activation of apoptotic pathway and cell death (39, 40). In addition to IRE1α, the other ER stress-driven UPR arms, involving PERK and ATF6, can also participate in MAM assembly and ER-mitochondria crosstalk. Indeed, ATF6 has been shown to promote mitochondrial biogenesis through interaction with peroxisome proliferator-activated receptor gamma coactivator 1α (PGC-1α) (41, 42), an effect that would presumably promote mitochondrial network remodeling and impact the ER-mitochondria interaction. PERK has also been reported to be located in the MAMs fraction and involved in mediating ER-mitochondria interaction during ROS-induced oxidative stress (43). Moreover, ATF4, located downstream of PERK, has been shown to regulate mitochondrial dynamics by controlling the expression of the ubiquitin ligase Parkin, that is responsible for the removal of the damaged mitochondria by mitophagy during ER stress (44). Interestingly, Parkin also mediates ER-mitochondria crosstalk during ER stress by increasing MAM microdomains assembly for maintaining intra-organelle Ca^2+^ transfer (45). In addition, ATF4 can participate to the regulation of the oxidative stress response and apoptosis in beta-cells through interaction with the transcription factor nuclear factor erythroid 2-related factor 2 (NRF2) (46–48). In fact, under inflammatory conditions, the increase in oxidative stress may lead to oxidation of cysteine residues on Kelch-like ECH associated protein 1 (KEAP1), leading to conformational changes and disruption of its interaction with NRF2. Next, NRF2 can translocate to the nucleus where it exerts a dual function. On one hand, it can form a heterodimer with ATF4 and regulate antioxidant genes (heme oxygenase-1, NAD(P)H:quinone oxidoreductase, glutathione S-transferase) and prevent cell death by activating anti-apoptotic genes (B-cell lymphoma-extra large (Bcl-xL), Bcl-2). On the other hand, it can also compete with ATF4 and inhibit its binding to the Amino Acid Responsive Element (AARE) on the CHOP promoter, ultimately limiting cytochrome C release and apoptosis (49).

Mitochondria form a very dynamic and plastic network that is continuously re-organized owing to tightly regulated processes involving fission, fusion and mitophagy-mediated clearance of dysfunctional organelles. On one hand, mitochondrial network remodeling, especially in beta-cell, is important for organelle bioenergetics and functions, including OXPHOS-driven ATP synthesis, Ca^2+^ signaling and cell survival. On the other hand, mitochondrial dynamics and their interaction with other subcellular structures are also regulated through intrinsic organelle metabolic activity and ROS production, with disrupted OXPHOS and increased oxidative stress promoting mitochondrial fission and mitophagy (50). In beta-cells, changes in the expression of mitochondrial fission/fusion proteins in response to both inflammatory and metabolic stresses have been shown to affect mitochondrial dynamic and ultrastructure, leading to impaired organelle energetics and altered beta-cell GSIS and survival (51). For instance, prolonged exposure to high glucose and/or lipotoxic environment increases the expression of the fission protein dynamin-related protein 1 (DRP1), reduces GSIS, and increases apoptotic cell death in INS-1E cells (52, 53). Similarly, DRP1 expression was found to be increased in beta-cells from dysfunctional pancreatic islets in a model of hyperglycemic T2D mice (54). In line with this, overexpression of DRP1 or FIS1 in various *in vitro* models of murine beta-cells was generally associated with increased mitochondrial fission, impaired mitochondrial bioenergetics and increased apoptosis (**Table S1**). More recently, the expression of the orphan nuclear receptor Nor1/Nr4a3, which was previously identified as a negative regulator of beta-cell mass (55), was found to be increased by proinflammatory cytokines and involved in beta-cell mitochondrial dysfunction (56). Indeed, following mitochondrial translocation, Nor1 promotes the disruption of mitochondrial network, leading to reduction in glucose oxidation and ATP production that ultimately contribute to apoptotic beta-cell death (56). By contrast, a balanced expression of the fission protein FIS1 was shown to be required for maintenance of mitochondrial network remodeling, beta-cell survival and efficient GSIS in INS-1 cells (57). Interestingly, dampening high glucose/lipid-induced beta-cell stress restores mitochondrial dynamics by increasing fusion and reducing fission, leading to decreased apoptosis in mouse islets (58).

## From Mitochondria Dysfunction to Beta-Cell Immunogenicity?

The prokaryotic origin of mitochondria positions the organelle as a potential source of intracellular mediators that can trigger innate immunity through release of various damage-associated molecular patterns (DAMPS) and/or pathogen-associated molecular patterns (PAMPS). Among these mitochondrial-derived molecules, specific phospholipids from the OMM (59), N-formyl methionine containing peptides which are legacy from prokaryotic translation initiation phase (60), and also mitochondrial DNA (mtDNA) containing hypomethylated CpG motifs that closely resemble bacterial CpG DNA can all trigger downstream immune activation, either directly or *via* pattern recognition receptor (PRRs)-mediated activation of the inflammasome.

In response to environmental stresses that lead to altered mitochondrial homeostasis, the organelle integrity is maintained, like in the ER, by chaperones (HSP60, HSP70) and proteases (ClpXP, ATP-dependent AAA protease LON) that control protein synthesis, folding and degradation and activate antioxidant mechanisms for ROS detoxification (61). Interestingly, the humoral and T cell reactivity against both HSP60 and HSP70 reported in newly diagnosed T1D patients may illustrate the important role played by mitochondrial dysfunction and stress in triggering autoimmunity (62, 63). During ER and/or mitochondrial stress, disruption in the Ca^2+^ homeostasis opens the mPTP which may lead to cytoplasmic release of mtDNA or mitochondrial double-stranded RNA (dsRNA) from mitochondria and induction of a programmed cell death. In line with this observation, it has been shown that activation of Bax/Bak during apoptosis led to the formation of a macropores on the OMM which allows the IMM to swell out into the cytosol and release mitochondrial factors, including mtDNA and dsRNA, without caspases activation (64). Classically, the presence of cytosolic mtDNA is sensed by cyclic GMP-AMP synthase (cGAS), resulting in stimulator of interferon genes (STING) activation and downstream phosphorylation of tyrosine kinase non receptor 1 (TNK1) and interferon regulatory factor 3 (IRF3), ultimately leading to increased transcription of type 1 IFN genes (65). Alternative pathways can trigger TLR9 activation within endosomes, leading to NFκB-mediated inflammasome (NLRP1 and NLRP3) activation, increased caspase 1 activity and subsequent IL-1β and IL-18 processing (66). Although little is known on the beta-cell inflammasome, studies conducted in both INS-1E cells and human islets have shown that prototypical inflammatory cytokines of the insulitis microenvironment may differentially regulate NLRP1 and NLRP3 *via* ATF4 and NFκB pathways (67). Similarly, cytosolic accumulation of dsRNA, generated after bidirectional transcription of circular mtDNA, can be detected by several cellular sensors, including protein kinase R (PKR), retinoic acid-inducible gene I (RIG-I) and melanoma differentiation-associated protein 5 (MDA5), to inhibit protein synthesis *via* phosphorylation of the eIF2α, recruitment of TANK-binding kinase-1, inducible IκB kinase (IKKϵ), and activation of IFN regulatory factor 3 and 7 and NFκB, to promote type I IFN secretion (68). Altogether, these findings highlight a potential mechanism by which ER stress-induced release of mitochondrial genomic material might lead to the activation of inflammatory pathways in beta-cells, contributing to their eventual demise.

The production of IL-1β, IL-18 or IFN-α/β by islet resident innate immune cells and/or endocrine cells (69–73), among which beta-cell is probably not the major source, facilitates the recruitment of immune cells [*e.g.* monocytes/macrophages, dendritic cells (DC)] to the inflammatory microenvironment within pancreas where they might contribute to the priming and activation of adaptive immune cells (74). Type I IFNs signature has been highly associated with disease development and progression from prediabetic stage (75). Moreover, among the 50 loci linked to T1D, several are expressed in beta-cells and involved in innate immunity [*e.g.* Protein Tyrosine Phosphatase, Non-Receptor Type 2 (PTPN2), Tyrosine Kinase 2 (TYK2), Interferon Induced Helicase C Domain 1 (IFIH1) and BTB Domain and CNC Homolog 2 (BACH2)]. In addition, a recent meta-analysis comparing RNAseq data from tissues of patients with T1D (beta-cells), systemic lupus erythematosus (SLE, kidney cells), multiple sclerosis (MS, optic chiasm) and rheumatoid arthritis (RA, joint tissue), identified a type I IFN signature as a common feature, further highlighting a more global role of type I IFN signaling in the development of autoimmune disease (76).

Although it is tempting to speculate that mitochondria permeability and subsequent genetic material leakage may participate to immune activation by beta-cells, strong supportive data are currently lacking to clearly implicate these processes in T1D development. However, a recent study has reported that inhibition of VDAC1 oligomerization during mitochondrial stress reduced accumulation of cytosolic mtDNA, expression of type I IFN genes, and circulating auto antibody levels in a mouse model of SLE, highlighting the possible role of cytosolic mtDNA in triggering autoimmunity (77). Furthermore, a relation between mitochondria content and beta-cell autoimmune destruction in T1D has also been suggested by the identification of a SNP located within the mitochondrial gene for NADH dehydrogenase 2 (*mt-Nd2*) that was associated with T1D (78). In this study, the presence of the *mt-Nd2a* (resistant) allele prevented both T1D after adoptive transfer of diabetogenic CD4+ T cell clones in NOD mice and beta-cell destruction by CD8 T cells *in vitro* (78). In addition, depletion of mtDNA from βlox5 cells lowered cytokine-mediated destruction and prevented CD8 T cell-mediated cytotoxic killing (79), positioning mtDNA a potentially important trigger of beta-cell destruction.

Alterations in intracellular Ca^2+^ homeostasis by ER/mitochondria stress may also have broad consequences on the cell visibility to the immune system by generating neoantigens, through regulation of mRNA splicing and protein synthesis or proteasomal degradation (80–82). Several studies have indeed highlighted the role of Ca^2+^ as activator for post translational enzymes transglutaminase 2 (TG2) and protein arginine deiminases (PADs) involved in deamidation and citrullination, respectively. These enzymes are mainly involved in coeliac disease and RA but have also been extensively studied in the context of T1D as important component of beta-cell-directed autoimmunity (3, 83, 84). In these processes, ROS participates in the regulation of post-translation modification (PTM) enzymes, inhibiting TG2 ubiquitination in endothelial cells or controlling activity of PAD4 (85, 86). Deamidation of glutamine and asparagine residues in the insulin B chain modifies the structural properties of insulin-derived epitopes and generates perfect anchors for peptide presentation in HLA-DQ2/-DQ8 predisposing haplotype (87). Similarly, citrullination has been shown to turn the GRP78/BiP ER chaperone into an important autoantigen in T1D by an ER stress-independent mechanism involving alteration of Ca^2+^ homeostasis and activation of PADs (84). In addition, changes in cytosolic Ca^2+^ level activate nuclear factor of activated T cells (NFAT) *via* the calcineurin/calmodulin pathway which plays a critical role in beta-cell proliferation and maintenance of beta-cell mass and function. As such, immunosuppressive drugs used in islet transplantation, such as cyclosporin A or FK506, inhibit calcineurin pathway, impair beta-cell function and trigger the development of post-transplantation diabetes mellitus (New Onset Diabetes After Transplantation [NODAT]) (88). Of note, new calcineurin inhibitors, such as voclosporin, has been recently reported to have less deleterious effects on islet functions (89). Increased cytosolic Ca^2+^ also promotes phospholipase C activation and production of IP3, which stimulates Ca^2+^ mobilization and subsequent activation of protein kinase C (PKC) and downstream MAP kinases (JNK and ERK) that ultimately leads to activation of NFκB-dependent transcriptional program and increased expression of the chemokines IL8, CCL2, CXCL10 and CXCL12 (25). Although Ca^2+^ chelation had serious deleterious effect on beta-cell function and GSIS *in vitro*, it prevented DC migration to insulinoma (90) and PAD enzymes activation (4), highlighting its role as a cellular mediator in the communication with the immune compartment. This also suggests that restoration of intracellular Ca^2+^ homeostasis may inhibit immune cell trafficking to the pancreatic islets and reduce beta-cell immunogenicity. Interestingly, chemical inhibition of the NFκB pathway reduced IL-8 production by stressed human beta-cells and prevented neutrophil migration, an effect mediated by the proton exchanger GRP68 and the transcription factor RFX6 which are also involved in the regulation of Ca^2+^ homeostasis (91).

Another consequence of ER stress and mitochondria dysfunction is the induction of cell senescence (92), secondary to increased ROS production and impaired redox status (21, 93, 94). Cellular senescence is a complex cell fate response that is characterized by the release of senescence-associated secretory phenotypes (SASPs) in response to multiple types of endogenous and exogenous stressors. The SASP components are of diverse nature, including cytokines and chemokines but also a large range of soluble and insoluble factors, and can contribute to immune activation, by promoting infiltration of immune cells. Although further studies are definitely required, it has been recently reported that islets from T1D mice as well as beta-cells from T1D donors display increased markers of senescence during disease progression (95), suggesting that beta-cell senescence may be an adaptive response to prolonged cellular stress that can contribute to autoimmunity through SASPs (96). Interestingly, studies aiming at eliminating senescent beta-cells by using specific senolytic drugs show remarkable results in limiting T1D progression in mice models (95, 97). These effects were associated with reduction in insulitis and improvements of both glucose metabolism and beta-cell function (95, 97). In response to metabolic activation and stress-associated UPRmt, mitochondria can also secrete mitochondria-derived peptides (MDPs), which are small bioactive peptides encoded by mtDNA that are mainly acting as retrograde signals to regulate mitochondrial energetics (98, 99). Although supporting data are currently sparse, especially in beta-cells, one can speculate that alterations in MDPs by inflammatory microenvironment may also contribute to modulate the cell communication with the immune compartment through various signaling pathways.

## Conclusion

Dysfunctional mitochondria has been particularly studied in the context of metabolic disorders and T2D, where it has been associated for decades to insulin resistance and beta-cell failure. Although the beta-cell failure in pancreatic islets differs in many aspect between T1D and T2D [for review (100)], the presence of an islet-specific inflammatory microenvironment characterized by elevated local concentrations of type 1 cytokines (67, 101), together with enhanced recruitment and/or activation of tissue-resident innate and adaptive immune cells (*e.g.* macrophages, B cells and T cells) (102) and accumulation of amyloid deposit (103, 104) represent common features to both pathologies. Consequently, the possibility of repurposing T2D drugs for T1D, to improve blood glucose management for relapsing metabolic and oxidative stress on beta-cells and limiting further immune destruction is worth considering. Ongoing studies using metformin, GLP-1 analogues, SGLT-2 inhibitors, or the L-VGCC inhibitor verapamil, which are either modulating mitochondrial bioenergetics/ROS/mPTP [metformin; (105–108)], ER stress [GLP-1 analogues; (109, 110)], glucose toxicity [SGLT-2 inhibitors; (111, 112)] or cellular Ca^2+^ homeostasis [verapamil; (113, 114)] are currently explored in the framework of clinical trials. These conceptually attractive novel approaches would also require further supportive mechanistic *in vitro* molecular studies in human primary beta-cells/islets and/or relevant beta-cell line models.

## Author Contributions

All the authors contributed equally to the review. SV and JL wrote the first draft of the manuscript. BG and AZ edited and wrote sections of the manuscript. AZ designed the figure. All authors contributed to the article and approved the submitted version.

## Funding

This work is supported by DON Foundation and the Dutch Diabetes Research Foundation, JDRF and by IMI2-JU under grant agreement No 115797 (INNODIA) and No 945268 (INNODIA HARVEST). This Joint Undertaking receives support from the Union’s Horizon 2020 research and innovation program and “EFPIA”, ‘JDRF” and “The Leona M. and Harry B. Helmsley Charitable Trust”.

## Conflict of Interest

The authors declare that the research was conducted in the absence of any commercial or financial relationships that could be construed as a potential conflict of interest.
