# Supplementary material for: Endoplasmic Reticulum-Mitochondria Crosstalk and Beta-Cell Destruction in Type 1 Diabetes
Source: Front Immunol. 2021 Apr 16;12:669492. doi: 10.3389/fimmu.2021.669492 (PMC8085402; doi:10.3389/fimmu.2021.669492)
Supplement: Supplementary file 1 [file Table_1.docx]

**Table S1. Impact of modulation of MAM components and proteins involved in mitochondrial dynamics on islets and/or beta-cells**

|  | **Protein** | **Model** | **Modulation** | **Phenotype** | **References** |
| --- | --- | --- | --- | --- | --- |
| **MAMs** | **IP3R** | MIN6 | Chemical activation | ↑ Thapsigargin-induced ER-stress and cell death | [1] |
|  |  | RINm5F | Chemical inhibition | ↓ IL-1β-mediated mitochondrial dysfunction and cell death | [2] |
|  |  | INS-1 | Chemical inhibition | ↓ ER Ca^2+^ depletion induced by cytokine stress | [3] |
|  |  | MIN6 | Chemical inhibition | ↓ Octanoic acid-induced GSIS | [4] |
|  | **GRP75** | INS-1 | Knockdown | ↓ ER-mitochondria interactions ↓ GSIS | [5] |
|  | **VDAC1** | INS-1 | Overexpression | ↓ Mitochondrial oxygen consumption ↓ GSIS | [6] |
|  |  | Human islets | Knockdown | ↑ Glucose oxidation and glucose-induced ATP production ↑ GSIS | [6] |
| **Mitochondrial dynamics** | **DRP1** | INS-1 | Overexpression | ↑ Mitochondrial fission | [7] |
|  |  | INS-1 | Doxycycline-inducible expression | ↑ Mitochondrial fission ↑ ROS ↑ Cytochrome C release  ↑ Caspase-3 activation ↑ High glucose-induced apoptosis | [8] |
|  |  | INS-1 | Doxycycline-inducible expression | ↓ Mitochondrial membrane potential ↑ ROS ↑ Cytochrome C release  ↑ Caspase-3 activation ↑ Lipid-induced apoptosis | [9] |
|  | **FIS1** | INS-1 | Overexpression | ↑ Mitochondrial fission ↓ Mitochondrial function and motility  ↓ Cellular ATP ↑ Apoptosis ↓ GSIS | [7] |
|  |  | RINm5F | Overexpression | **Moderate expression**: ↑ GSIS  **High expression**: ↑ Mitochondrial size ↓ GSIS | [10] |
|  |  | INS1-832/13 | Overexpression | ↓ Mitochondrial size ↑ GSIS | [10] |
|  |  | Primary mouse beta-cells | Overexpression | ↑ Mitochondrial elongation ↓ GSIS | [10] |
|  |  | INS1-832/13 | Knockdown | ↑ Mitochondrial size ↓ GSIS | [10] |
|  |  | Primary mouse beta-cells | Knockdown | ↑ Mitochondrial fragmentation ↓ GSIS | [10] |
|  |  | INS-1 | Knockdown | ↓ Mitophagy ↑ Oxidized mitochondrial proteins  ↓ Mitochondrial respiration ↓ ROS ↓ GSIS | [11] |
|  |  | INS-1 | Knockdown | ↓ Mitochondrial fission ↓ Apoptosis | [12] |
|  | **OPA1** | Primary mouse beta-cells  INS-1 | Overexpression | **Moderate expression**: ↑ Mitochondrial fusion  **High expression**: ↑ Mitochondrial fission | [12] |
|  |  | INS-1 | Overexpression | ↓ Mitophagy | [11] |
|  |  | Mouse islets | Knockdown | ↑ Mitochondrial fission ↓ Respiration ↓ Glucose-induced ATP production ↓ GSIS | [13] |
|  | **MFN1**  **MFN2** | INS-1 cells | Overexpression | ↑ Mitochondrial fusion ↓ Mitochondrial motility and function | [14] |
|  |  | INS-1 cells | Overexpression | ↑ Mitochondrial fusion ↓ Cellular ATP ↓ GSIS | [7] |
|  |  | C57BL/6J mice | Pancreas-specific MFN1/2 Knockout | ↑ Mitochondrial fission ↓ Mitochondrial respiration and membrane potential  ↓ Glucose-induced ATP production ↓ GSIS ↓ Beta-cell mass | [15] |
|  |  | Balb/c mice | siRNA MFN2 knockdown | ↓ GSIS | [16] |

**References**

[1] D.S. Luciani, K.S. Gwiazda, T.L. Yang, T.B. Kalynyak, Y. Bychkivska, M.H. Frey, K.D. Jeffrey, A.V. Sampaio, T.M. Underhill, and J.D. Johnson, Roles of IP3R and RyR Ca2+ channels in endoplasmic reticulum stress and beta-cell death. Diabetes 58 (2009) 422-32.

[2] G. Verma, H. Bhatia, and M. Datta, JNK1/2 regulates ER-mitochondrial Ca2+ cross-talk during IL-1beta-mediated cell death in RINm5F and human primary beta-cells. Mol Biol Cell 24 (2013) 2058-71.

[3] W.R. Yamamoto, R.N. Bone, P. Sohn, F. Syed, C.A. Reissaus, A.L. Mosley, A.B. Wijeratne, J.D. True, X. Tong, T. Kono, and C. Evans-Molina, Endoplasmic reticulum stress alters ryanodine receptor function in the murine pancreatic beta cell. J Biol Chem 294 (2019) 168-181.

[4] J. Leem, H.M. Shim, H. Cho, and J.H. Park, Octanoic acid potentiates glucose-stimulated insulin secretion and expression of glucokinase through the olfactory receptor in pancreatic beta-cells. Biochem Biophys Res Commun 503 (2018) 278-284.

[5] E. Tubbs, P. Theurey, G. Vial, N. Bendridi, A. Bravard, M.A. Chauvin, J. Ji-Cao, F. Zoulim, B. Bartosch, M. Ovize, H. Vidal, and J. Rieusset, Mitochondria-associated endoplasmic reticulum membrane (MAM) integrity is required for insulin signaling and is implicated in hepatic insulin resistance. Diabetes 63 (2014) 3279-94.

[6] E. Zhang, I. Mohammed Al-Amily, S. Mohammed, C. Luan, O. Asplund, M. Ahmed, Y. Ye, D. Ben-Hail, A. Soni, N. Vishnu, P. Bompada, Y. De Marinis, L. Groop, V. Shoshan-Barmatz, E. Renstrom, C.B. Wollheim, and A. Salehi, Preserving Insulin Secretion in Diabetes by Inhibiting VDAC1 Overexpression and Surface Translocation in beta Cells. Cell Metab 29 (2019) 64-77 e6.

[7] K.S. Park, A. Wiederkehr, C. Kirkpatrick, Y. Mattenberger, J.C. Martinou, P. Marchetti, N. Demaurex, and C.B. Wollheim, Selective actions of mitochondrial fission/fusion genes on metabolism-secretion coupling in insulin-releasing cells. J Biol Chem 283 (2008) 33347-56.

[8] X. Men, H. Wang, M. Li, H. Cai, S. Xu, W. Zhang, Y. Xu, L. Ye, W. Yang, C.B. Wollheim, and J. Lou, Dynamin-related protein 1 mediates high glucose induced pancreatic beta cell apoptosis. Int J Biochem Cell Biol 41 (2009) 879-90.

[9] L. Peng, X. Men, W. Zhang, H. Wang, S. Xu, Q. Fang, H. Liu, W. Yang, and J. Lou, Involvement of dynamin-related protein 1 in free fatty acid-induced INS-1-derived cell apoptosis. PLoS One 7 (2012) e49258.

[10] J. Schultz, R. Waterstradt, T. Kantowski, A. Rickmann, F. Reinhardt, V. Sharoyko, H. Mulder, M. Tiedge, and S. Baltrusch, Precise expression of Fis1 is important for glucose responsiveness of beta cells. J Endocrinol 230 (2016) 81-91.

[11] G. Twig, A. Elorza, A.J. Molina, H. Mohamed, J.D. Wikstrom, G. Walzer, L. Stiles, S.E. Haigh, S. Katz, G. Las, J. Alroy, M. Wu, B.F. Py, J. Yuan, J.T. Deeney, B.E. Corkey, and O.S. Shirihai, Fission and selective fusion govern mitochondrial segregation and elimination by autophagy. EMBO J 27 (2008) 433-46.

[12] A.J. Molina, J.D. Wikstrom, L. Stiles, G. Las, H. Mohamed, A. Elorza, G. Walzer, G. Twig, S. Katz, B.E. Corkey, and O.S. Shirihai, Mitochondrial networking protects beta-cells from nutrient-induced apoptosis. Diabetes 58 (2009) 2303-15.

[13] Z. Zhang, N. Wakabayashi, J. Wakabayashi, Y. Tamura, W.J. Song, S. Sereda, P. Clerc, B.M. Polster, S.M. Aja, M.V. Pletnikov, T.W. Kensler, O.S. Shirihai, M. Iijima, M.A. Hussain, and H. Sesaki, The dynamin-related GTPase Opa1 is required for glucose-stimulated ATP production in pancreatic beta cells. Mol Biol Cell 22 (2011) 2235-45.

[14] K.S. Park, A. Wiederkehr, and C.B. Wollheim, Defective mitochondrial function and motility due to mitofusin 1 overexpression in insulin secreting cells. Korean J Physiol Pharmacol 16 (2012) 71-7.

[15] E. Georgiadou, C. Muralidharan, M. Martinez, P. Chabosseau, A. Tomas, F. Yong Su Wern, T. Stylianides, A. Wretlind, C. Legido-Quigley, N. Alsabeeh, C. Cruciani-Guglielmacci, C. Magnan, M. Ibberson, I. Leclerc, Y. Ali, A.K. Linnemann, T.A. Rodriguez, and G.A. Rutter, Mitofusins Mfn1 and Mfn2 are required in the β-cell to preserve mitochondrial architecture and insulin secretion. BioRxiv (2020).

[16] L. Li, Z.F. Pan, X. Huang, B.W. Wu, T. Li, M.X. Kang, R.S. Ge, X.Y. Hu, Y.H. Zhang, L.J. Ge, D.Y. Zhu, Y.L. Wu, and Y.J. Lou, Junctophilin 3 expresses in pancreatic beta cells and is required for glucose-stimulated insulin secretion. Cell Death Dis 7 (2016) e2275.
